# The Effect of Copper–Graphene Composite Architecture on Thermal Transport Efficiency

**DOI:** 10.3390/ma16227199

**Published:** 2023-11-17

**Authors:** Arseny M. Kazakov, Galiia F. Korznikova, Ilyas I. Tuvalev, Artem A. Izosimov, Elena A. Korznikova

**Affiliations:** 1 Research Laboratory “Metals and Alloys under Extreme Impacts”, Ufa University of Science and Technology, 450076 Ufa, Russia; 2 Institute of Metal Superplasticity Problems (IMSP), 450001 Ufa, Russia; 3 Department of Surgical Dentistry, Bashkir State Medical University, 450008 Ufa, Russia; 4 The World-Class Advanced Digital Technologies Research Center, Peter the Great St. Petersburg Polytechnic University, 29 Polytechnicheskaya Str., 195251 St. Petersburg, Russia; 5 Technological Machines and Equipment Department, Ufa State Petroleum Technological University, 450064 Ufa, Russia

**Keywords:** copper, graphene, composite, molecular dynamics method, thermal conductivity

## Abstract

This paper presents the results of molecular dynamic modeling, revealing that inserting confined graphene layers into copper crystal reduces the thermal conductivity of the whole composite, and the coefficient of thermal conductivity *κ* decreases upon an increase in the number of graphene layers. The injection of one, two, and three layers of 15 nm graphene leads to a change in the coefficient of thermal conductivity from 380 W/(m·K) down to 205.9, 179.1, and 163.6 W/(m·K), respectively. Decreasing the length of graphene layers leads to a decrease in the density of defects on which heat is dissipated. With one, two, and three layers of 8 nm graphene, the coefficient of thermal conductivity of the composite is equal to 272.6, 246.8, and 240.8 W/(m·K), appropriately. Meanwhile the introduction of an infinite graphene layer results in the growth of *κ* to 414.2–803.3 W/(m·K).

## 1. Introduction

The development of novel composites that exhibit improved mechanical properties by incorporating carbon structures and metal nanoparticles is of great importance. In recent decades, extensive research efforts have been focused on the integration of graphene with some of the most commonly used metals, such as aluminum, nickel, copper, titanium, and silver [1,2,3,4].

Among these metals, copper (Cu) has garnered significant attention, mainly due to its excellent thermal conductivity and cost-effectiveness [5]. The study of copper–graphene composites holds great significance, as it offers a versatile material with high thermal conductivity, mechanical strength, and electrical properties. This makes it suitable for a wide range of applications, including efficient thermal management in smart electronics and the development of high-strength conductive materials and next-generation conductors [4,5,6].

Furthermore, the incorporation of graphene has been found to mitigate the thermal expansion coefficient in comparison to pure copper, a characteristic particularly advantageous for electronic applications [7,8,9].

An important result of this research is the suggestion of using copper–graphene composites as coatings [10,11,12], thanks to their enhanced tribological properties. Additionally, within a graphene network, copper matrix composites have exhibited an impressive combination of high strength and ductility [13,14,15].

Recent research in the field of copper–graphene composites focuses on improving the stability of three-dimensional samples. The investigation actively explores the structure, strength, corrosion resistance, and tribological properties for potential applications across various industrial sectors [16,17,18]. However, there is limited research on how graphene affects the thermal conductivity of these composites. For instance, in one study [19], the authors explore the possibility of adding copper–graphene composites to epoxy resins to enhance thermal conductivity. Another study [20] discusses the deposition of graphene on the surface of copper, which was treated with laser cladding. This process significantly improves the thermal conductivity of the resulting material. Additionally, new methods are under investigation to produce copper–graphene composites without degrading their electrical conductivity properties [21]. Given the high cost of conducting experiments with graphene, computer modeling is a cost-effective approach for research. Both first principles and molecular dynamics (MD) modeling methods have been used to study graphene properties [22,23]. MD requires fewer computational resources and is, therefore, more affordable, providing results that correlate well with the first principles method. Moreover, the MD method is versatile and can be applied to study various processes in different areas, such as the transformation of defect systems in crystal lattices [24] and the thermal stability of reinforced carbon nanotubes [25]. In the context of graphene, MD has been successfully used to examine its behavior in different graphene-based composites and their mechanical properties [26,27,28,29,30,31].

The architecture of copper–graphene composites plays a crucial role in achieving a balance between increasing thermal conductivity through a new transport channel that covers a broad frequency range and dissipating heat at newly established interfaces. In many studies that explore how graphene enhances the thermal conductivity of metallic materials, the internal architecture of the composite is often overlooked. More specifically, the involvement of graphene in the transport process and the establishment of contact between graphene and the heat source are frequently neglected [30]. The primary aim of this study was to investigate various structures of copper–graphene composites to gain insights into the key factors influencing the complex thermal properties of the material. This work, in particular, presents simulation results regarding the impact of graphene on the coefficient of thermal conductivity in copper–graphene composites, with a focus on the number of graphene layers, their length, and their positioning at room temperature.

## 2. Materials and Methods

The simulation was carried out using a free package for classical molecular dynamics Large-scale Atomic/Molecular Massively Parallel Simulator (LAMMPS) [32], and The Open Visualization Tools program was used to visualize the simulation results [33].

**Figure 1:**
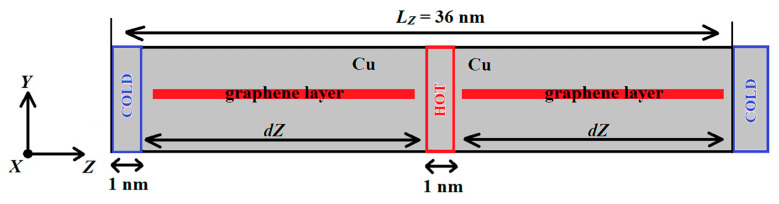
The scheme of the simulation cell with the temperature block positions and edges of periodic boundary conditions along *Z* axis.

The calculation cell was a single crystal of fcc copper, with graphene layers introduced in the middle part. The linear dimensions of the calculated cells are approximately 4.7 × 4.7 × 36 nm^3^, and the total number of atoms in the cells are around 67,000. Periodic boundary conditions were applied along all three directions. The Langevin thermostat was used to heat limited blocks of the crystal and calculate the thermal conductivity of the composite with the timestep of 0.2 fs. Thermal conductivity calculation time is equal to 1 ns. The temperatures of hot and cold blocks were 320 K and 280 K, respectively. The heat from the hot block to the cold block spread along the *Z* axis. The length of the blocks was equal to 1 nm. Figure 1 shows the scheme of the simulation conditions. The simulation cell contains only one cold block (which is in the left) and one hot block in the middle; therefore there is no symmetry in the cell. This positioning of the blocks is imposed due to periodic boundary conditions.

To calculate the thermal conductivity of Cu–graphene composite, the non-equilibrium method suggested by Ikeshoji and Hafskjold was used [34]. The following formulas were used in the calculations:

(1) $$\kappa =dQ*\frac{dZ}{dT},$$

where *κ* is the coefficient of thermal conductivity, *dQ* is the energy flux, and *dT*/*dZ* is the temperature gradient (as the heat spreads along the *Z* axis).

(2) $$dQ=\frac{N*(Q_{1}-Q_{2})}{2*n*\tau *S*2}.$$

In this formula, *N* is the number of atoms, (*Q*_1_ − *Q_2_*)/2 is the average total of input and output energy for two regions normalized by atoms, *n* is the number of steps, *τ* is the timestep, *S* is the *XY* box area, *dT* is the average temperature difference between two regions, and *dZ* is the distance between hot and cold blocks. The division by 2 should be taken into account, since energy flux goes in two directions due to periodic boundary conditions. Using these formulas, *κ* was calculated for all of the considered cases.

**Figure 2:**
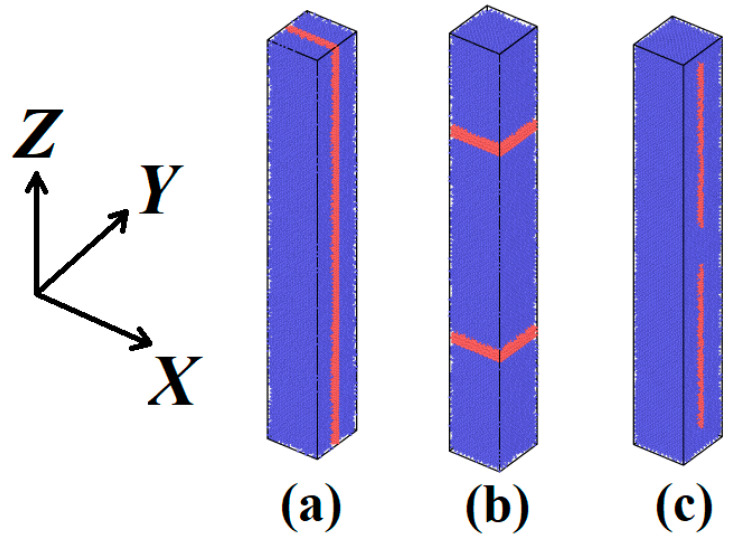
Types of all studied cases with infinite graphene layers along (**a**) and perpendicularly (**b**) to the *Z* axis and copper-capsuled graphene layers (**c**).

One, two, or three layers of graphene with varying lengths were introduced into the simulation cell at specific positions. For the Cu–graphene system, it was essential to select potentials capable of accurately describing the covalent bonds within graphene, the interaction between Cu atoms and graphene, and the interactions among Cu atoms. We utilized the AIREBO potential [35] for graphene–graphene interactions, the EAM potential developed by Zhou [36] for Cu–Cu interactions, and the Morse potential with parameters *D_e_* = −0.100 eV, *R_e_* = 2.220 Å, *β* = 1.700 1/Å for Cu–graphene interactions. The cutoff distance for Cu–graphene interactions was set at 6.5 Å. The choice of these parameters has previously been successfully validated in earlier research [37]. Figure 2 depicts the simulation cells with different configurations of graphene layers relative to copper.

In this work, the following cases were studied: one, two, or three graphene layers relative to copper. These configurations included infinite graphene layers along the *Z* axis (Figure 2a), infinite graphene layers perpendicular to the *Z* axis (Figure 2b), and copper-encapsulated graphene layers with lengths of 15 nm and 8 nm along the *Z* axis (Figure 2c). The thermal conductivity of all the aforementioned cases was compared to that of a pure copper crystal with the same geometry.

## 3. Results and Discussion

**Figure 3:**
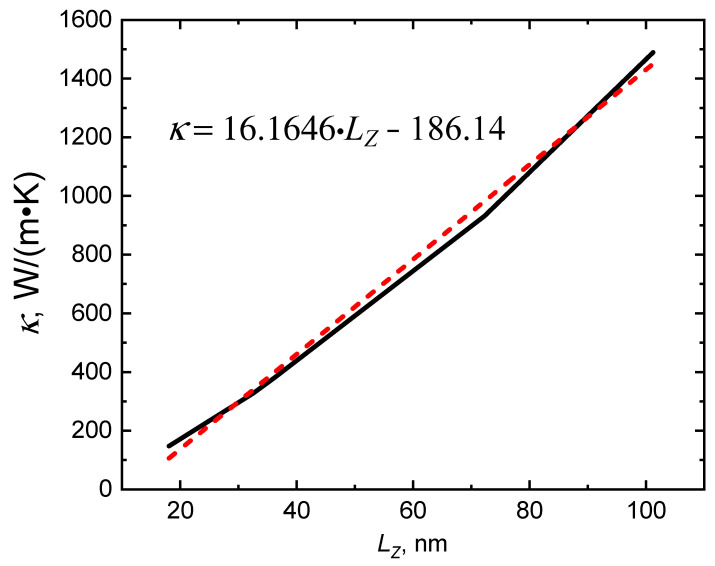
Dependence of coefficient of thermal conductivity *κ* on cell size *L_Z_* for pure Cu crystal. The red dashed line represents the linear dependence. The figure has been updated.

Firstly, it is important to note that the coefficient of thermal conductivity is influenced by the size of the simulation cell, as demonstrated by Formula (2). To determine the dependence, a curve of the coefficient of thermal conductivity values on the length of the simulation cell size *L_Z_* was obtained for pure Cu crystal, which is shown in Figure 3. In order to get the most accurate results for the effect of graphene on composite thermal conductivity, the value of *L_Z_* = 36 nm was chosen, and in our simulations, the coefficient of thermal conductivity *κ* for pure Cu crystal was equal to 380 W/(m·K). This value coincides with the tabulated value of the thermal conductivity of copper. The size-dependent behavior of the coefficient of thermal conductivity can be attributed to what is known as ballistic conduction. Ballistic conduction pertains to the conduction of electrons in a material with minimal scattering, resulting in a notably high level of conductivity [38]. This phenomenon occurs when the mean free path of electrons, the distance they can travel without significant scattering, surpasses the minimum dimension of the sample. Ballistic conduction is frequently observed in quasi-one-dimensional structures like carbon nanotubes or silicon nanowires, primarily due to the pronounced size quantization effects in such materials [39]. It should be noted that the linear approximation of this dependence in the form of the equation *ax + b* has an error for *a* in the range of 4% and for *b* in the range of 20%.

**Figure 4:**
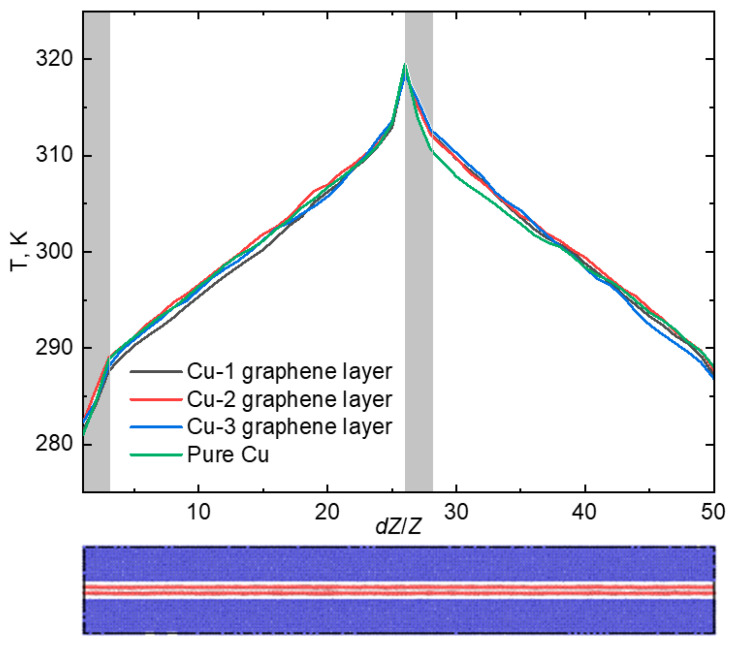
The temperature gradient for the case of infinite graphene along *Z* axis. The gray areas show the operating zones of Langevin thermostat.

Scenarios involving infinite layers of graphene were investigated, and, in line with previous cases, the thermal conductivity calculations were performed over an extended time frame of 1 ns. This extended time frame was chosen to enhance accuracy and gather statistical data. The temperature gradient along the *Z* axis is illustrated in Figure 4.

This distribution of temperature across the simulation cell was presented to show the influence of applied simulation conditions such as the thermostat operation zone and the graphene positioning on the thermal conductivity properties of the composites.

It is evident from the chart that the temperature aligns in all three scenarios with the temperature of pure Cu crystal. The simulation cell size *L_Z_* was partitioned into 50 sections, and the temperature value was recorded at every time step. Following 1 ns of calculation time, the temperature was averaged. As previously stated, the coefficient of thermal conductivity (*κ*) for pure Cu crystal equaled 380 W/(m·K). Upon introducing one graphene layer, *κ* rose by 9%, reaching 414.2 W/(m·K). The coefficient of thermal conductivity of the composite with two layers of graphene is 654.6 W/(m·K). Once three layers of graphene had been inserted into the Cu crystal, its thermal conductivity increased by more than twice the amount when compared with pure Cu—resulting in a value of *κ* at 803.3 W/(m·K). As can be observed, an increase in the number of graphene layers within the composite resulted in a rise in the coefficient of thermal conductivity. This is due to the overlapping of the phonon spectrums of Cu and graphene. In [40], the authors analyzed the phonon dispersion curves of graphene grown on a single-crystal metal surface. The focus was specifically on the interaction between graphene and Cu, as although weak, clear signatures were detected in the phonon dispersion curves.

Interestingly, the coefficient of thermal conductivity for a single layer of graphene was determined to be 390 W/(m·K). However, when a monolayer of graphene was incorporated into copper, the composite exhibited higher thermal conductivity than the monolayer of graphene alone. In contrast, when two or three layers of graphene were added to copper, the resulting thermal conductivity of the composites was lower than that of the individual graphene layers. This is evidenced by the coefficient of thermal conductivity values of 729.3 W/(m·K) and 1128.3 W/(m·K) for two and three layers of graphene, respectively, as discussed in the previous section. Furthermore, when comparing the thermal conductivity of graphene and copper with identical geometric parameters, it was revealed that graphene had a coefficient of thermal conductivity of 4921.1 W/(m·K), nearly 13 times higher than that of pure copper crystal.

**Figure 5:**
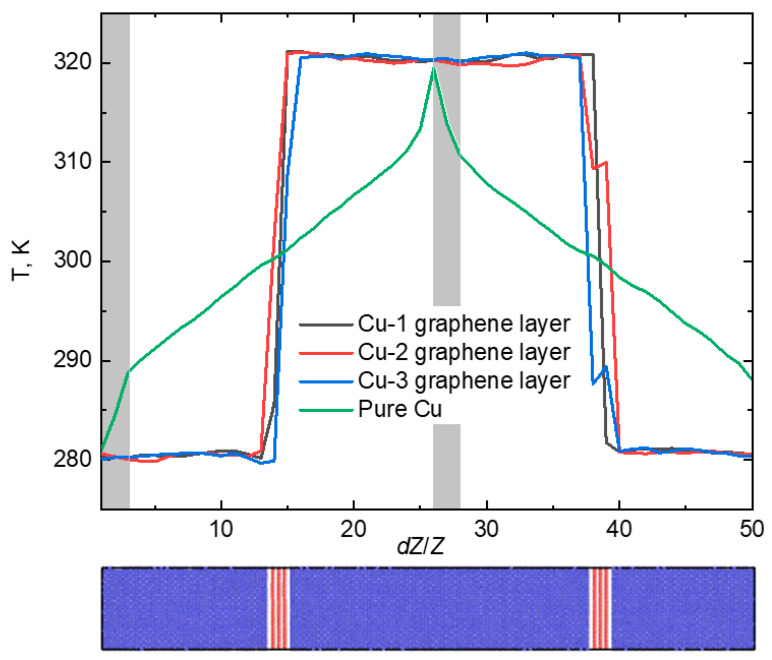
The temperature gradient for the case of infinite graphene perpendicular to *Z* axis. The gray areas show the operating zones of Langevin thermostat.

In the second case, the positioning of the graphene layers is oriented perpendicular to the *Z* axis. The temperature gradient in this scenario is illustrated in Figure 5.

Figure 5 demonstrates that graphene layers effectively act as barriers to the propagation of heat, primarily due to the well-established phenomenon of heat dissipation on defects [41]. Consequently, the center of the crystal retains a higher temperature, while the edges remain relatively cooler. As heat does not readily spread along the crystal, it was expected that the coefficient of thermal conductivity for the composites would be low. Indeed, the thermal conductivity (*κ*) values for composites containing one, two, and three layers of graphene are 2.51, 2.18, and 1.53 W/(m·K), respectively. Graphene, aligned along the path of heat propagation, absorbs phonons at the interface and does not effectively conduct heat, instead releasing heat energy.

**Figure 6:**
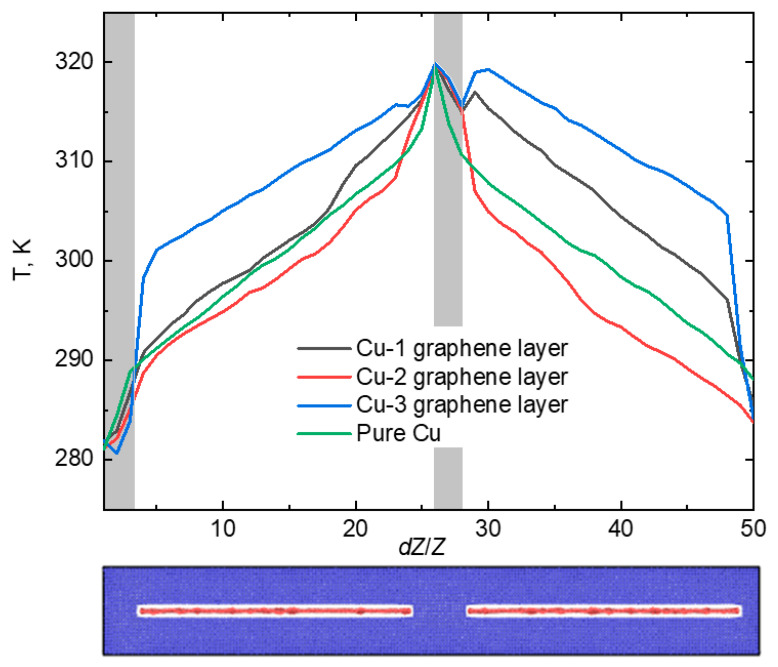
The temperature gradient for the case of copper-capsuled graphene along *Z* axis with the length of 15 nm. The gray areas show the operating zones of Langevin thermostat.

The next step involved examining the scenarios that incorporated copper-capsulated graphene layers with lengths of 15 and 8 nm. As shown in Figure 6, the temperature gradient was displayed for the composites that included the 15 nm graphene layers.

As shown in Figure 6, the temperature distribution along the crystal exhibits a similar shape, but the composite with three graphene layers heats up much more than the other cases, while the composite with two graphene layers heats less than the pure Cu crystal and the composite with one graphene layer. However, the values of κ change monotonously. For the composite with one graphene layer, it equals 205.9 W/(m·K), with two graphene layers, it decreases to 179.1 W/(m·K), and for the crystal with three graphene layers, *κ* = 163.6 W/(m·K). This behavior can be explained by the fact that the graphene–copper interfaces act as defects that dissipate heat when encountered. A study [42] delved into the impact of an increasing number of graphene layers on the thermal conductivity of the interface, which aligns with our findings. As the number of graphene layers in the crystal increases, the number of defects also increases, and it is natural that the thermal conductivity will deteriorate. Even though the thermal conductivity of graphene surpasses that of copper, the influence of defects is of greater significance. The uneven temperature distribution may be attributed to the proximity of graphene layers to both cold and hot regions.

**Figure 7:**
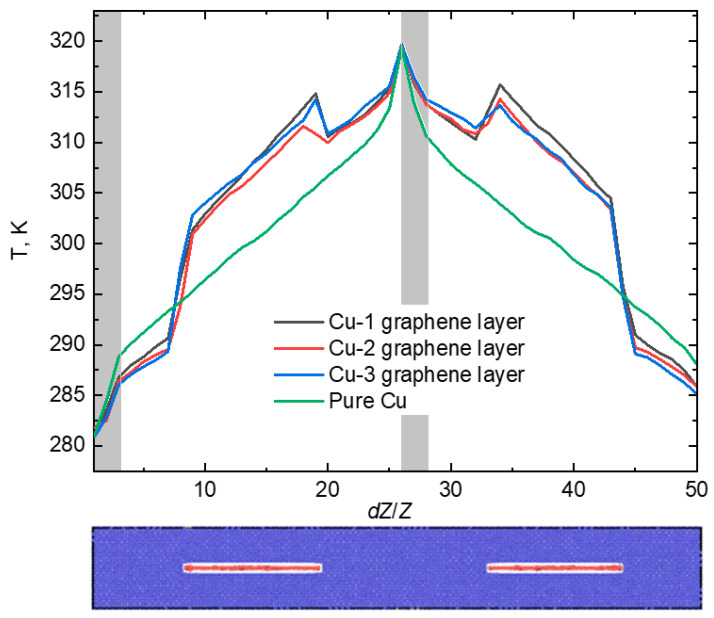
The temperature gradient for the case of copper-capsuled graphene along *Z* axis with a length of 8 nm. The gray areas show the operating zones of Langevin thermostat.

In order to investigate the effect of the length of graphene layers, let us look at the behaviour of 8 nm graphene layers and compare it with the previous case. Figure 7 shows the temperature gradient for the composites with 8 nm graphene layers.

**Figure 8:**
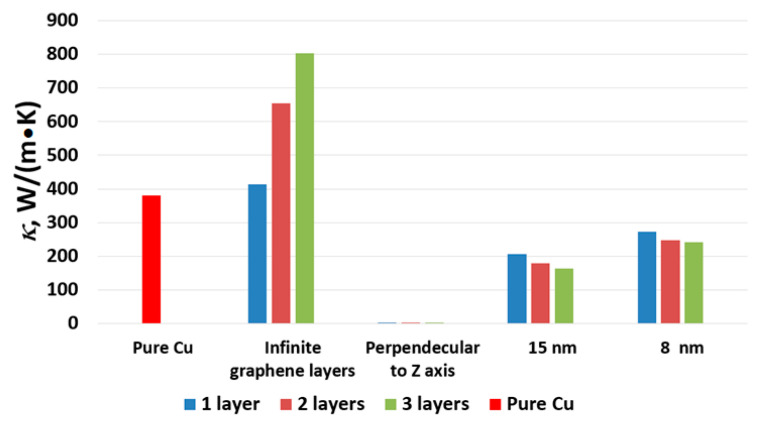
Histogram with the values of the coefficient of thermal conductivity *κ* for all considered cases. Number of layers is colored according to legend.

In Figure 7, it is evident that localized heating occurred on the graphene layers within all composites. The graph exhibited distinct steps, reflecting the temperature behavior of each structural component of the crystal, including the hot and cold blocks, Cu, and graphene. Based on Figure 7, it can be concluded that the values of *κ* for the composites were expected to be similar. Indeed, the coefficient of thermal conductivity for the composite with one graphene layer was 272.6 W/(m·K), for the composite with two graphene layers, it was 246.8 W/(m·K), and for the crystal with three graphene layers, it measured 240.8 W/(m·K). As observed in the previous case with 15 nm graphene layers, thermal conductivity decreased with an increase in the number of graphene layers. The reason remained consistent—an increase in the number of graphene layers led to an increase in the number of defects. The influence of graphene layer length on composite thermal conductivity was apparent and could be explained in the same manner. Table 1 displays the values of the coefficient of thermal conductivity (*κ*) for Cu–graphene composites, and Figure 8 provides a visual representation of the obtained values to facilitate the visualization of the effect of graphene layers on thermal conductivity.

The coefficient of thermal conductivity of the composite increases only in the scenario where the graphene layers are infinite along the *Z* axis. Conversely, a reduction in thermal conductivity is observed for other cases, particularly for copper-encapsulated graphene layers of 15 nm and 8 nm. Furthermore, it is important to note that all examined composites exhibit favorable thermal stability, with no significant alterations detected during the calculations.

**Figure 9:**
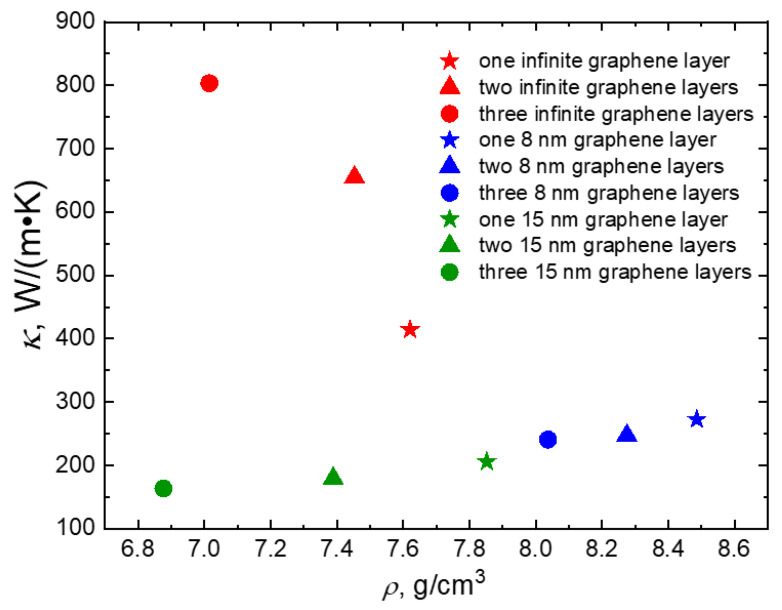
The dependence of coefficient of thermal conductivity *k* on composite density *ρ*. In the figure, different composite configurations are denoted by color, and the number of graphene layers are represented by the star (one layer), triangle (two layers), and circle (three layers) for infinite layers case (red), 8 nm layers case (blue), and 15 nm layers case (green), respectively.

When examining various cases with different composite configurations, it is crucial to understand how the coefficient of thermal conductivity varies with composite density. The composite density values were estimated using a mass-to-volume ratio approximation. The relationship between the heat transfer coefficient and composite density is depicted in Figure 9. In this analysis, we did not consider the scenario where the graphene layers are perpendicular to the *Z* axis because this hinders heat conduction rather than facilitating it.

As depicted in Figure 9, the density of the composite decreases as the number of graphene layers increases. For composites with copper–graphene layers, there is an almost linear increase in the coefficient of thermal conductivity as composite density rises. However, in the case of infinite graphene layers along the *Z* axis, an increase in composite density leads to a decrease in the coefficient of thermal conductivity. This behavior can be explained as follows: when graphene is influenced by the Langevin thermostat, it heats up more rapidly than in other scenarios, resulting in an increase in the coefficient of thermal conductivity. Describing the dependence of the coefficient of thermal conductivity on composite density is challenging here, as computational cell dimensions and the operational zones of the Langevin thermostat have a significant impact on this process.

In addition to all of the above, the analytical approach for predicting the coefficient of thermal conductivity was tested for the all considered cases. The thermal conductivity of copper composites can be calculated using the following equation:

(3) $$k’=\sum k_{i}V_{i}$$

where *k′* is the thermal conductivity of the composite, *k_i_* is the thermal conductivity of the ith component, and *V_i_* is the volume fraction of ith component [43]. The values of graphene volume fraction and its coefficient of thermal conductivity *k^Gr^* are presented in Table 2.

Once again, only three of the configuration cases with the same thermal conductivity mechanism are discussed, excluding the case where graphene layers act as a barrier. It is worth noting that technical term abbreviations are explained upon first use. We can obtain the values of the coefficient of thermal conductivity *k′* by substituting the values from Table 2 and the known value of the Cu coefficient of thermal conductivity *κ^Cu^* = 380 W/(m·K) into Equation (3), and these values are collected in Table 3.

Upon comparing Table 1 and Table 3, it is clear that there is a good qualitative convergence and numerical consistency among the results. This is because Equation (3) does not consider the configurations of the composites; thus, it cannot account for the graphene–copper interfaces where heat is dissipated. The significant variation in the coefficients of thermal conductivity between cases with an indefinite number of graphene layers may be attributed to the positioning of the graphene within the operating zones of the Langevin thermostat, which has a significant impact on *κ*.

In summary, there is no unified strategy or model currently available to comprehensively describe the thermal properties of metal–graphene composites, taking into account all their components and structural features. Nevertheless, the strong nonlinear interactions of graphene enhance the heat conduction efficiency in a system by introducing additional phonon modes. This effect is most pronounced in copper–diamond composites, where the high stiffness and oscillation frequencies of the carbon component lead to a significant increase in thermal conductivity [44]. However, this effect is somewhat attenuated when graphene is used, as it has a lower phonon velocity range compared to diamond [45]. This weakening can also be observed when calculating thermal conductivity for a composite with an infinite sheet of graphene, as shown in Figure 2a. Initially, heat transfer takes place across all frequencies, leading to increased efficiency, which offsets the reduction in the *κ* coefficient resulting from heat dissipation at boundaries, ultimately resulting in increased thermal conductivity. However, in scenarios where heating occurs only in a portion of the copper-bound sheet from the outset, the frequencies supported by this lattice realize the enhanced heat transfer capability of graphene, making the material less efficient. At the same time, the aforementioned heat dissipation effect at the boundaries leads to a reduction in κ compared to pure copper.

## 4. Conclusions

In this work, the influence of the length and number of graphene layers on thermal conductivity of a copper–graphene composite was studied at room temperature within the use of a molecular dynamics method.

The obtained results provide valuable insights into the engineering of graphene–metal composites. When analyzing composites with infinite graphene sheets, it becomes clear that efficiency initially increases when heat transfer occurs throughout the material. However, in cases where heating is confined to a portion of the copper-bounded sheet, efficiency decreases, influenced by heat dissipation at boundaries. This complexity underscores the challenges involved in comprehending the thermal characteristics of these composites.

We believe that this study can contribute to a better understanding of the interaction between copper and graphene, as well as the development of copper–graphene composites with enhanced thermal conductivity properties. Future studies will focus on investigating the thermal conductivity of copper–graphene composites after undergoing severe plastic deformation, such as equal channel angular pressing or high-pressure torsion, which will allow for the assessment of a broader range of defect configurations contributing to the thermal properties of graphene.

## Figures and Tables

**Table 1:** The values of the coefficient of thermal conductivity *κ* for all considered cases.

*κ*, W/(m·K)					
Number of Graphene Layers	Pure Cu Crystal	Inf. Graphene Layers along the *Z* Axis	Inf. Graphene Layers Perpendicularly to the *Z* Axis	15 nm Graphene Layers	8 nm Graphene Layers
1	380	414.2	2.51	205.9	272.6
2		654.6	2.18	179.1	246.8
3		803.3	1.53	163.6	240.8

**Table 2:** The values of graphene volume fraction and coefficient of thermal conductivity for all considered cases.

	Volume Fraction			*k^Gr^*, W/(m·K)		
Number of Graphene Layers	Inf. Graphene Layers along the *Z* Axis	15 nm Graphene Layers	8 nm Graphene Layers	Inf. Graphene Layers along the *Z* Axis	15 nm Graphene Layers	8 nm Graphene Layers
1	0.104	0.085	0.047	390.0	82.1	26.2
2	0.154	0.128	0.066	729.3	153.5	49.0
3	0.208	0.188	0.095	1128.3	237.5	75.8

**Table 3:** The values of the coefficient of thermal conductivity *κ*′ for all considered cases.

*Κ*′, W/(m·K)			
Number of Graphene Layers	Inf. Graphene Layers along the *Z* Axis	15 nm Graphene Layers	8 nm Graphene Layers
1	381.1	354.7	363.4
2	433.6	353.2	358.2
3	535.6	351.0	351.1

## Data Availability

The datasets are available from the corresponding author upon reasonable request.
